# Narrative microstructure and macrostructure in adolescents with Down syndrome and Williams syndrome

**DOI:** 10.3389/fpsyg.2025.1402121

**Published:** 2025-01-28

**Authors:** Aitana Viejo, Maite Fernández-Urquiza, Eliseo Diez-Itza

**Affiliations:** LOGIN Research Group, University of Oviedo, Oviedo, Spain

**Keywords:** Down syndrome, Williams syndrome, intellectual disabilities, genetic syndromes, atypical language profiles, pragmatic assessment, narrative microstructure and macrostructure

## Abstract

Down syndrome (DS) and Williams syndrome (WS) are genetic neurodevelopmental disorders associated with intellectual disability, showing contrasting linguistic profiles with asymmetries in grammatical (DS weakness/WS strength) vs. pragmatic abilities (DS strength/WS weakness). The aim of the present study was to explore the linguistic profiles of 14 adolescents with DS and WS, and 14 typically developing controls (matched by chronological and verbal age) by comparing the microstructure and macrostructure of narratives and their possible dissociation. Participants watched an episode of the Tom and Jerry cartoon series and were asked to retell it. The videotaped narratives were transcribed and analyzed with the tools of the CHILDES Project and the Pragmatic Evaluation Protocol for Corpora (PREP-CORP). Microstructure was assessed by productivity at the grammatical level (number of utterances) and lexical level (number of word tokens), and complexity at the grammatical level (MLU) and lexical level (number of word types). Macrostructure was assessed by the number of story elements recalled at three levels: scenarios (global), episodes (integrated), and events (detailed). Results confirmed asymmetries in the linguistic profiles of both groups, with relative strengths of adolescents with DS in macrostructure despite relative weaknesses in microstructure. Conversely, adolescents with WS exhibited strengths in narrative microstructure, but failed to show better performance than the DS group in macrostructure. Following regression analyses, microstructure predicted macrostructure in typically developing adolescents, while no association was found between both levels in the profiles of adolescents with WS and DS, which was interpreted as an atypical dissociation.

## 1 Introduction

Down syndrome (DS) and Williams syndrome (WS) are genetic neurodevelopmental disorders that cause intellectual disability and have distinct behavioral phenotypes (Antonarakis et al., 2020; Kozel et al., 2021). Regarding their linguistic profiles, specific opposing strengths and weaknesses have been described: grammatical weakness vs. pragmatic strength in DS, and grammatical strength vs. pragmatic weakness in WS (Abbeduto et al., 2016).

Several studies have confirmed special difficulties in expressive language in DS, particularly in syntactic complexity (Abbeduto et al., 2003; Chapman, 2003; Chapman et al., 1998; Chapman and Hesketh, 2000; Diez-Itza and Miranda, 2007; Fowler, 1990; Miller, 1999; Roberts et al., 2007; Vicari et al., 2000). In turn, WS has been characterized by a special grammatical strength (Bellugi et al., 1988, 2000; Brock, 2007; Mervis, 2006; Mervis et al., 2003), although it was overestimated by comparisons with DS; a number of studies point out that this strength is relative and not necessarily obvious when compared to mental age or chronological age matched controls (Diez-Itza et al., 2017; Diez-Itza et al., 2019; Joffe and Varlokosta, 2007; Stojanovik et al., 2004).

The study of narrative competence in both syndromes is one of the main sources of evidence about relative strengths and weaknesses at grammatical and pragmatic levels. The analysis of narratives has been conducted in modern studies through the assessment of microstructure (i.e., grammatical and lexical productivity and complexity) and macrostructure (i.e., episodic structure, characters, story grammar) and the relative strengths and weaknesses at each level. The results of these studies have been interpreted in terms of possible dissociations, asymmetries and asynchronies in the linguistic profiles of the syndromes which have been an important source for the comprehension of neurodevelopmental disorders (Karmiloff-Smith, 1998; Thomas et al., 2009).

Pioneering studies of children and adolescents with DS focused on syntactic and lexical production in narrative microstructure, observing fewer utterances and lower mean length of utterance (MLU) and lexical diversity than typically developing (TD) controls matched by mental, syntactic and lexical age (Chapman et al., 1990; Hesketh and Chapman, 1998).

Subsequent analyses of narrative macrostructure found that DS narratives were longer and structurally more complex than those of their MLU-matched TD controls (Boudreau and Chapman, 2000); such relative strength in macrostructure was further confirmed by Miles and Chapman (2002) who found that children and adolescents with DS outperformed their MLU-matched TD controls when narrating the main plot, the story outline, and the adventures of the characters. However, Kay-Raining Bird et al. (2008) found that children with DS matched by reading skills with TD controls produced longer narratives, but with similar linguistic complexity and episodic structure.

More recently, Channell et al. (2015) confirmed relative strengths in the macrostructure as children and adolescents with DS narrated the story structure at the level of non-verbal mental age TD controls, despite a general impairment in expressive grammar and a specific deficit in the production of verbs. Based on the retelling of a picture story book, Zanchi et al. (2021) also found that children and adolescents with DS produced stories comparable to those of TD children at both the macrostructural and microstructural levels, except for syntactic complexity. Conversely, Martzoukou et al. (2020) explored narrative skills in adults with DS, who presented relative weaknesses in lexical diversity and grammatical complexity but they also produced less content related to the story structure than their TD controls matched by expressive vocabulary; in the same vein, Mattiauda et al. (2022) also reported that adults with DS scored below their vocabulary matched TD controls on measures of story structure and story comprehension, as well as lexical diversity.

Research on narrative competence in WS has included in some cases DS control groups. That was the case in the first study by Reilly et al. (1990), who observed greater coherence and narrative complexity in the narratives of adolescents with WS than in DS controls. Losh et al. (2000) found a higher proportion of morphological errors and lower syntactic complexity in children with WS than in chronological age and gender-matched TD controls. In a longitudinal study, Reilly et al. (2004) observed that children with WS acquired the morphology and syntax of English at the same rate and level as controls with a diagnosis of Specific Language Impairment (SLI). However, when comparing the groups on narrative measures, children in the WS group were consistently delayed in aspects which tap cognitive skills such as making inferences about characters’ relationships and motivations throughout the story and integrating the local episodic elements with the more global theme of the story. From these “divergent profiles” they concluded that language develops independently of other cognitive abilities in both groups, and that in the WS group structural language is a relative strength contrasting with a weakness in the integration and inferencing in the narratives linked to their lower IQ.

Studies in languages other than English confirm atypical characteristics in WS, with specific strengths in grammatical skills, but weaknesses in structural coherence and complexity of the narrative process, suggesting asymmetries and dissociations between microstructure and macrostructure (Antón et al., 2007; Diez-Itza and Miranda, 2005; Diez-Itza et al., 2016, 2018; Garayzábal et al., 2007; Gonçalves et al., 2004, 2010; Lacroix et al., 2007; Marini et al., 2010; Perovic et al., 2024; Reilly et al., 2005; Shiro et al., 2019).

Studies of narrative competence used different procedures for eliciting narratives and analyzing the components of microstructure and macrostructure: the more widespread is a procedure based on the wordless picture book “Frog, where are you?” by Mayer (1969) usually named “The Frog Story” (Channell et al., 2015; Garayzábal et al., 2007; Gonçalves et al., 2004, 2010; Kay-Raining Bird et al., 2008; Lacroix et al., 2007; Losh et al., 2000; Reilly et al., 1990, 2004, 2005; Miles and Chapman, 2002); the elicitation of personal narratives and the description of pictures or photographs (Chapman et al., 1990; Diez-Itza and Miranda, 2005; Hesketh and Chapman, 1998; Marini et al., 2010; Zanchi and Zampini, 2021); the retelling of oral narratives using the *Multilingual Assessment Instrument for Narratives* (Martzoukou et al., 2020); or the retelling of a wordless film (Antón et al., 2007; Boudreau and Chapman, 2000; Diez-Itza et al., 2016), which is the method used in the present study, where the narrative structure is not given verbally to the subjects, and therefore it may better replicate the cognitive task of constructing a mental model of the story involved in narrative competence (Bruner, 1991; Stein and Glenn, 1982).

In the context of the SYNDROLING Project (Diez-Itza et al., 2014), a procedure developed by Diez-Itza et al. (2001) based on the retelling of a wordless film, namely an episode of the Tom and Jerry cartoon series, was used to elicit the narratives; in addition, a Pragmatic Evaluation Protocol for Corpora was developed (PREP-CORP; Fernández-Urquiza et al., 2017) as a tool for tagging pragmatics in speech corpora based on a previous clinical protocol (PREP; Gallardo-Paúls, 2009). The original PREP and the PREP-CORP include three levels of pragmatic analysis (enunciative, textual and interactive); the present study focuses on textual pragmatic items to analyze the macrostructure of the narratives based on the sequence of events considered at three levels: single events (complex/detailed), events grouped in episodes (intermediate/integrated), and events taking place in scenarios (basic/global) (Diez-Itza et al., 2018; Shiro et al., 2019).

We use these procedures in the present study to address the research question of the existence of possible asymmetries and dissociations in the narrative profiles of individuals with neurodevelopmental disorders. The objective then is to explore the narrative profiles in adolescents with DS and WS, based on the analyses of the microstructure (productivity and complexity) and the macrostructure (levels of organization). The literature has pointed out that linguistic profiles of both syndromes are uneven, so the first hypothesis is that the profiles will present asymmetries in the form of strengths and weaknesses; it has also been discussed if microstructure and macrostructure could be dissociated, so a second hypothesis would be that, if the profiles are uneven, this will yield a dissociation between microstructure and macrostructure.

## 2 Method

### 2.1 Participants

The study included 28 participants divided into four groups of 7 Spanish-speaking participants each (3 males/4 females): a group of adolescents with DS; a TD control group matched to the DS group by verbal age (MLU); a group of adolescents with WS; and a TD control group matched to the WS group by chronological age. MLU was calculated based on the number of words per utterance in the narratives. The participants were selected from a larger group within the SYNDROLING Project (Diez-Itza et al., 2014) and had been diagnosed by the genetic services of the Central University Hospital of Asturias (HUCA). All of them or their legal tutors signed an informed consent. Table 1 shows the chronological and verbal age of the participants.

**Table 1:** Chronological and verbal age of the participants.

	DS Mean (SD) Range	WS Mean (SD) Range	TD-CA Mean (SD) Range	TD-VA Mean (SD) Range
CA	17.03 (1.42) 15.58–19.83	21.32 (2.97) 18.08–26.10	21.25 (3.21) 18.32–26.60	3.58 (0.36) 3.08–4.08
VA (MLU)	4.88 (1.32) 2.71–6.76	8.28 (2.44) 4.94–13.03	11.34 (1.52) 9.16–13.11	4.91 (1.32) 3.28–6.64

CA, chronological age; VA, verbal age; MLU, mean length of utterances; DS, Down syndrome; WS, Williams syndrome; TD-CA, chronological-age-matched control group; TD-VA, verbal-age-matched control group; SD., standard deviation.

### 2.2 Procedure

Narratives were elicited from each participant after viewing an episode of the “Tom & Jerry” cartoon series (*The Puppy Tale*). They were instructed to retell the cartoon to the researcher while being videotaped. Narrative corpora were transcribed and analyzed with the tools of The CHILDES Project (CLAN programs) (MacWhinney, 2000) and the PREP-CORP Protocol (Fernández-Urquiza et al., 2017). Coding was conducted independently by two researchers, and both analyzed the entire sample, while conflicting cases were solved by a third researcher to reach 100% agreement. They used as “gold standard” a coding scheme which analyzes the macrostructure at three levels: (i) scenarios: basic or global level, corresponding to the locations or spaces in which the initiating event, complication, high point, and resolution of the story took place; (ii) Episodes: intermediate or integrated level, corresponding to sets of actions whose sequencing constitute the plot of the story; (iii) Events: complex or detailed level, corresponding to the sequence of single actions making up the story (see Diez-Itza et al., 2018, pp. 7–8).

### 2.3 Data analyses

The microstructure of the narratives was analyzed based on the following variables: productivity (grammatical: number of utterances; lexical: number of word tokens) and complexity (grammatical: MLU; lexical: number of word types). The macrostructure variables of the study were: global level (4 scenarios), integrated level (10 episodes), and detailed level (25 events).

Shapiro–Wilk and Levene tests were used to confirm statistical normality and homoscedasticity. To assess group differences, one-way ANOVAs and HSD Tukey *post hoc* tests were conducted. Effect sizes (*d* Cohen and *r*) were calculated following Lenhard and Lenhard (2022). Furthermore, linear and logarithmic regression analyses were performed to assess relationships between microstructure and macrostructure.

## 3 Results

Table 2 reports means and standard deviations of microstructure (utterances, tokens, MLU, and types) and macrostructure (scenarios, episodes, and events) variables, as well as the results of the one-factor ANOVAs performed to assess differences between groups. Differences were found in all the variables both at the microstructure and macrostructure, with large effect sizes.

**Table 2:** Comparisons of means (ANOVAs) and effect size for microstructure and macrostructure variables.

		**DS-G** Mean (SD)	**VA-G** Mean (SD)	**WS-G** Mean (SD)	**CA-G** Mean (SD)	***F***	***p***	***d***	***r***
Microstructure	UTT	29.1 (10.1)	16.3 (5)	32.4 (10.1)	26.4 (9.5)	4.291	0.015	1.196	0.513
	TOK	140.6 (53.9)	80.1 (27.3)	259.6 (86.9)	302.1 (123.8)	11.197	0.000	1.932	0.694
	MLU	4.9 (1.3)	4.9 (1.3)	8.3 (2.4)	11.3 (1.5)	22.970	0.000	2.767	0.810
	TYP	64.7 (24.8)	44 (12.8)	103.7 (26.3)	124.6 (34.3)	14.119	0.000	2.169	0.735
Macrostructure	SCN	3.4 (0.5)	2.1 (0.9)	3.6 (0.5)	3.7 (0.5)	9.059	0.000	1.738	0.655
	EPS	5.6 (1.4)	3.3 (1.3)	7 (2.2)	8.9 (1.7)	13.660	0.000	2.134	0.729
	EVT	8.4 (2.2)	4.4 (1.3)	10.7 (4)	16.6 (4.9)	15.408	0.000	2.266	0.749

G, group; DS, Down syndrome; WS, Williams syndrome; CA, chronological age; VA, verbal age; SD, standard deviation; *d*, Cohen’s *d*; UTT, number of utterances; TOK, number of tokens; MLU, mean length of utterances; TYP, number of types; SCN, scenarios; EPS, episodes; EVT, events.

Table 3 shows mean differences between groups and the results of the post-hoc Tukey’s HSD test. Statistical differences between the syndromic groups corresponded to lower grammatical (MLU) and lexical (word types) complexity in DS group, but no differences were further observed in grammatical (utterances) and lexical (word tokens) productivity and in the macrostructure variables.

**Table 3:** *Post-hoc* analysis (HSD Tukey) of mean differences in microstructure and macrostructure.

		DS – WS (*d*)	DS – VA (*d*)	DS – CA (*d*)	WS – VA (*d*)	WS – CA (*d*)	CA – VA (*d*)
Microstructure	UTT	−3.3 (0.33)	12.9 (1.62)	2.7 (0.28)	16.1* (2.04)	6 (0.61)	10.1 (1.34)
	TOK	−119 (1.65)	60.4 (1.42)	−161.6** (1.69)	179.4** (2.79)	−42.6 (0.75)	222*** (2.48)
	MLU	−3.4** (1.73)	0 (0)	−6.5*** (4.54)	3.4** (1.72)	−3.1* (1.51)	6.4*** (4.52)
	TYP	−3.9* (1.53)	20.7 (1.05)	−59.9*** (1.99)	59.7*** (2.89)	−20.9 (0.68)	80.6*** (3.11)
Microstructure	SCN	−0.1 (0.27)	1.3** (1.74)	−0.3 (0.56)	1.4** (1.93)	−0.1 (0.28)	1.6*** (2.17)
	EPS	−1.4 (0.77)	2.3 (1.72)	−3.3** (2.13)	3.7** (2.05)	−1.9 (0.94)	5.6*** (3.77)
	EVT	−2.3 (0.71)	4 (2.21)	−8.1*** (2.13)	6.3* (2.12)	−5.9* (1.31)	12.1*** (3.37)

DS, Down syndrome; WS, Williams syndrome; CA, chronological age; VA, verbal age; * *p* < 0.05; **p < 0.01; ***p < 0.001; UTT, number of utterances; TOK, number of tokens; MLU, mean length of utterances; TYP, number of types; SCN, scenarios; EPS, episodes; EVT, events; *d*, Cohen’s *d*, effect size.

Adolescents with DS did not differ from their verbal-age controls in the microstructure, but they did recall a greater number of scenarios at the global level of the macrostructure. Compared to chronological-age controls, they showed lower lexical productivity (word tokens) and lower grammatical (MLU) and lexical complexity (word types) in the microstructure, as well as lower recall at the levels of episodes and events in the macrostructure.

The adolescents with WS showed greater productivity and complexity in the microstructure, and greater recall of all levels of the macrostructure than the children in the VA-TD group, but they exhibited lower grammatical complexity (MLU) in microstructure and lower recall of the detailed level (events) in macrostructure than their CA-TD controls. The children in the VA-TD group had lower values in all variables than the adolescents in the CA-TD group, except for number of utterances (see Table 3).

To analyze the relationships between microstructure and macrostructure, simple regression analyses were conducted, taking microstructure variables (utterances, tokens, MLU and types) as independent variables. In the CA-TD group and, to a lesser extent, in the VA-TD group, model adjustments to curvilinear (logarithmic) regression models were observed. Conversely, in the groups of adolescents with DS and with WS, regression models failed to predict macrostructure from microstructure variables (see Table 4).

**Table 4:** Logarithmic regression model for CA-TD controls.

IV	DV	*R* ^2^	*F*	*p*	Ct	b1
UTT	SCN	0.791	18.913	0.007*	0.179	1.101
	EPS	0.754	15.319	0.011*	−3.001	3.691
	EVT	0.918	55.712	0.001*	−21.892	11.974
TOK	SCN	0.620	8.173	0.035*	−1.203	0.873
	EPS	0.648	9.186	0.029*	−8.399	3.063
	EVT	0.847	27.645	0.003*	−41.449	10.299
MLU	SCN	0.001	0.003	0.956	3.937	−0.092
	EPS	0.008	0.040	0.849	6.228	1.086
	EVT	0.041	0.212	0.665	−0.930	7.230
TYP	SCN	0.728	13.352	0.015*	−3.086	1.420
	EPS	0.701	11.696	0.019*	−14.066	4.786
	EVT	0.802	20.268	0.006*	−55.546	15.057

IV, independent variable; DV, dependent variable; Ct, constant; SCN, scenarios; EPS, episodes; EVT, events; UTT, number of utterances; TOK, number of tokens; MLU, mean length of utterances; TYP, number of types; *p < 0.05.

Table 4 shows adjustments to curvilinear (logarithmic) regression models in the group of CA-TD controls. Microstructure variables, except for grammatical complexity (MLU), explain high proportions of the variances observed at all macrostructure levels. In the case of VA-TD controls, adjustments to logarithmic regression models were only observed in grammatical (utterances) and lexical (word tokens) productivity: number of utterances predicted recall of scenarios (*R*^2^ = 0.595, *F* = 7.342, *p* = 0.042, Ct = −2.767, b1 = 1.795) and of episodes (*R*^2^ = 0.777, *F* = 17.395, *p* = 0.009, Ct = −4.531, b1 = 2.857); while number of word tokens predicted recall of events (*R*^2^ = 0.603, *F* = 7.585, *p* = 0.040, Ct = −3.663, b1 = 1.884).

## 4 Discussion

The aim of the present study was to explore the narrative competence of adolescents with Down syndrome (DS) and William syndrome (WS), comparing them with two groups of typically developing (TD) participants matched by verbal and chronological age. We analyzed the microstructure (productivity: number of utterances and word tokens; complexity: MLU and number of word types) and the macrostructure (scenarios, episodes, and events) of narratives elicited from the retelling of a cartoon episode, using the tools of the CHILDES Project (MacWhinney, 2000) and the PREP-CORP Protocol (Fernández-Urquiza et al., 2017). Furthermore, possible dissociations between microstructure and macrostructure were explored through regression analyses.

All participants in the study were able to narrate the story, and accordingly whole measurements of productivity and complexity of the microstructure and of macrostructure levels were obtained. Thus, the narrative analysis methodology proved to be feasible for the naturalistic assessment of pragmatic competence in neurodevelopmental disorders (Barokova and Tager-Flusberg, 2018). Specifically, the PREP-CORP Protocol (Fernández-Urquiza et al., 2017) provided a practical coding system at the level of textual pragmatics as previous studies had revealed (Diez-Itza et al., 2018; Shiro et al., 2019).

### 4.1 Narrative microstructure

The length of the narratives (number of utterances) of the adolescents with DS and WS was comparable to that of their CA-TD controls, indicating special strengths in grammatical productivity (i.e., utterances) of the microstructure in both syndromes. No differences were found in lexical productivity (i.e., word tokens) between the syndromic groups, although special strength might only be attributed to the WS group since the adolescents with DS produced fewer word tokens than their TD peers.

In the WS group, the strengths observed in grammatical and lexical productivity were as expected since WS has been characterized by its especially preserved language (Bellugi et al., 1988, 2000; Brock, 2007; Mervis et al., 2003). Conversely, strength in grammatical productivity was not expected in the DS group, since verbal production has been described as an area of relative weakness in this syndrome when considering non-verbal cognitive level (Abbeduto et al., 2003; Chapman et al., 1998; Chapman and Hesketh, 2000; Vicari et al., 2000). Specifically, the narratives produced by adolescents with DS were found to be shorter than those of Fragile X syndrome (FXS) and TD controls matched by mental and lexical age (Channell et al., 2015; Chapman et al., 1990; Kover et al., 2012). However, different studies have found relative strengths in the grammatical productivity of individuals with DS. Chapman et al. (1998) observed a greater production of utterances but a lower MLU in the narratives of children and adolescents with DS when compared to TD controls matched by non-verbal mental age; Kay-Raining Bird et al. (2008) also found longer narratives in adolescents with DS than in TD children matched by reading level even though controls had better lexical comprehension. Enhanced production of shorter utterances in narratives of adolescents with DS could be explained by their fair understanding of the story schema while lacking enough linguistic competence to narrate the plot in a more elaborated way. In the same vein, Del Hoyo Soriano et al. (2020) observed a longitudinal increase in talkativeness and a decrease in grammatical complexity (i.e., MLU) and lexical complexity (i.e., word types) in adolescents with DS and FXS.

Those findings are consistent with the results of the present study showing lower grammatical and lexical complexity in the DS group than in the WS group, suggesting that it is in complexity rather than in productivity where lies the particular weakness in expressive language displayed by individuals with DS (Chapman, 2003; Chapman and Hesketh, 2000; Diez-Itza and Miranda, 2007; Fowler, 1990; Miller, 1999). Their development of grammatical complexity is seriously limited by the tendency to omit grammatical morphemes (Chapman et al., 2002; Diez-Itza and Miranda, 2007; Fowler et al., 1994). Expressive language is usually below receptive language and what would be expected for non-verbal mental age (Diez-Itza et al., 2019; Laws and Bishop, 2003; Martin et al., 2013). Some studies indicate that weakness is more pronounced in syntax than in vocabulary, but others based on the analysis of narratives did not observe more weakness in grammatical complexity than in lexical complexity, in line with the results of the present study (Chapman et al., 1990, 1998; Finestack and Abbeduto, 2010; Keller-Bell and Abbeduto, 2007; Martzoukou et al., 2020).

The narratives of the WS adolescents presented shorter utterances than those of their CA-TD controls, thus grammatical complexity (MLU) was the only variable in the microstructure that showed relative weakness, which contrast with the special strength observed in lexical complexity (types). These results are consistent with previous studies that opposed the hypothesis of grammar preservation in WS, as discussed in Diez-Itza et al. (2017, 2019). Conversely, the observed lexical strength is consistent with the specific profiles of WS described in many studies that emphasize the breadth of their vocabulary, especially of concrete words (Abbeduto et al., 2016; Bellugi et al., 1990, 2000; Kozel et al., 2021; Mervis and Becerra, 2007; Mervis and John, 2008).

Overall, the analysis of narrative microstructure revealed that the syndromic linguistic profiles are not homogeneous: in DS, the strength in grammatical productivity (i.e., utterances) did not correspond to greater length (MLU) and lexical diversity (types) of utterances; in WS, grammatical complexity (i.e., MLU) was lower than expected considering grammatical productivity (i.e., utterances) and lexical complexity (i.e., types). The narrative profiles of both syndromes would present specific asymmetries different from typical development, and thus suggesting asynchronous developmental trajectories (Levy and Eilam, 2013; Karmiloff-Smith, 2007).

The observed asymmetries also shed light on the debate about estimating verbal age from expressive syntax (MLU) and lexical comprehension (Channell et al., 2015; Miles et al., 2006). The MLU-based verbal age matching used in many studies may underestimate grammatical (utterances) and lexical (tokens) productivity of adolescents with DS and WS, and lexical complexity (types) in the latter (Miles et al., 2006; Thordardottir et al., 2002). On the other hand, vocabulary test scores would provide a better estimate of verbal age (Diez-Itza et al., 2019). In any case, the results of the present study highlight the need to assess narrative production using different measures and from naturalistic language samples (Abbeduto et al., 2020; Adams, 2002; Barokova and Tager-Flusberg, 2018).

### 4.2 Narrative macrostructure

Adolescents in the syndromic groups did not differ at any level of the narrative macrostructure: global (scenarios), integrated (episodes), and detailed (events), suggesting relative pragmatic strength in the adolescents with DS, despite their grammatical and lexical difficulties. They also outperformed the VA-TD controls at the global level of macrostructure, i.e., retelling as the CA-TD controls most of the scenarios of the story, which also underscored such strengths in DS and is consistent with previous studies (Boudreau and Chapman, 2000; Channell et al., 2015; Finestack et al., 2012; Hogan-Brown et al., 2013; Kay-Raining Bird et al., 2008; Miles and Chapman, 2002). However, the same strengths are not present at the integrated (episodes) and detailed (events) levels, which also suggests macrostructure asymmetries in the DS narrative profile.

In contrast to their strengths in microstructure, adolescents with WS displayed relative weakness at the detailed level of macrostructure (recall of events), which is consistent with most previous studies on narrative competence reporting weaknesses in story structure (Garayzábal et al., 2007; Gonçalves et al., 2010; Lacroix et al., 2007; Marini et al., 2010; Reilly et al., 2004); but it is not consistent with the results of our previous research, indicating relative strength in the detailed level of events (Diez-Itza et al., 2002, 2006), which could be explained by the use of a different story (“Frog goes to dinner” film) and inclusion of both children and adult participants with WS in those studies. However, adolescents with WS in the present study exhibited particular strengths at the integrated (recall of episodes) and global (recall of scenarios) levels, revealing again asymmetries in the macrostructure. In this regard, atypical language profiles and nonlinear trajectories of relative strengths and weaknesses that may change with age have been observed in WS (Diez-Itza et al., 2017, 2019; Pérez et al., 2022).

Relative weakness of adolescents with WS when narrating story details would not correspond with their tendency to local processing in visuospatial construction tasks (Bihrle et al., 1989; Diez-Itza et al., 2016; Mervis, 2006). In children with WS, alterations have also been observed in visual perception of motion stimuli and in spatial localization memory (Atkinson et al., 1997; Nakamura et al., 2001), which are similar to those exhibited by individuals with Autistic Spectrum Disorder (ASD) in dynamic spatial processing tasks (Bertone et al., 2005; Milne et al., 2002). In contrast to WS and ASD, individuals with DS show global processing strengths in visuospatial construction tasks and, therefore, the adolescents with DS in the present study may have benefited from the visual support implicit in the elicitation method based on the recall of a cartoon (Bertone et al., 2005; Miles et al., 2006).

The asymmetries and differences found in the macrostructure could also be related to the ability to understand and construct a “mental model” of the story and to organize events sequentially and causally (Bruner, 1991; Diez-Itza et al., 2016; Garnham et al., 1982; Stein, 1982), that might be affected in different ways by the intellectual disability of the adolescents in the syndromic groups. Reilly et al. (2004) attributed the weakness in narrative structure of children with WS to their cognitive impairment, even though they showed better linguistic performance than their SLI controls. The cognitive processing profile in WS has been associated with that observed in ASD, including dissociations between structural and figurative language linked to weak central coherence, and alterations in the organization and causal coherence of narratives that have been reported in both neurodevelopmental disorders (Capps et al., 2000; Happé and Frith, 2006; Norbury et al., 2014; Gillam et al., 2015; Vulchanova et al., 2015).

### 4.3 Relations between microstructure and macrostructure

Regression analyses models failed to establish relationships between narrative microstructure and macrostructure in the syndromic groups, in contrast to what was observed in the TD groups. In the CA-TD group, logarithmic regression models were adjusted for grammatical (utterances) and lexical (tokens) productivity and lexical complexity (types) in the microstructure, explaining high proportions of the variance at all levels of the macrostructure. In the VA-TD group, model adjustments were only observed for productivity of utterances predicting global and integrated levels of the macrostructure, and for productivity of word tokens predicting detailed levels, which suggests that the relationships between microstructure and macrostructure change with age, pointing to emergent non-linear and dynamic trajectories of progressive functional integration (Karmiloff-Smith, 2009).

Only grammatical complexity (MLU) did not predict any of the levels of the macrostructure, that is, reduced MLU did not convey less elaborated levels of narrative macrostructure in either the syndromic groups or their TD controls, as already observed by Diez-Itza et al. (2018). For this reason, the adolescents with DS did not differ from those with WS on retelling the macrostructure of the story, although their utterances were shorter, suggesting that MLU may not be a good predictor of pragmatic skills and presents limitations as a matching variable, since it may change depending on the task (Miles et al., 2006). In contrast, lexical comprehension has been found to be the best predictor of narrative skills in DS (Kay-Raining Bird et al., 2008).

The CA-TD controls who produced more word tokens mentioned more events, but those who presented a greater diversity of vocabulary in their narratives showed better recall of story plot episodes and the global framework of story scenarios. This could suggest that lexical productivity (tokens) would be more related to the recall of story details, while lexical complexity (types) would be more related to the ability to construct the story schema at the cognitive level (Bruner, 1990, 1991; Stein and Glenn, 1982), which would be in line with studies that have linked vocabulary to cognitive level (Jensen, 2001; Smith et al., 2005). The special strengths in productivity (utterances and tokens) and lexical complexity (types) in participants with WS did not determine a better retelling of narrative macrostructure than those in the DS group, suggesting again atypical dissociations between language and cognition in WS, beyond the deficit in spatial cognition previously reported (Atkinson et al., 1997; Bihrle et al., 1989; Nakamura et al., 2001). Visuospatial cognition had been related to vocabulary characteristics in the WS group, with concrete vocabulary being a relative strength that justifies the observed dissociation; in contrast, relational vocabulary referring to more abstract concepts, which form the basis for the cognitive construction of narrative schemas, is very limited and is at the level of visuospatial construction ability (Mervis and John, 2008).

A number of limitations of the present study need to be acknowledged before drawing any conclusions: although the observed differences yielded large effect sizes, the sample size was small and sex differences were not assessed, hence the results can only be interpreted as exploratory; as mentioned above, MLU may not be an appropriate matching variable, so future studies could include lexical verbal age for that purpose; the emphasis on group mean differences and similarities may have obscured important individual differences that are generally present in neurodevelopmental disorders (Stojanovik et al., 2006); the correlations between cognitive and linguistic abilities observed in previous studies suggest that the absence of control groups matched for mental age or memory measures may have prevented ruling out the effect of these variables, yet it remains a controversial issue (Karmiloff-Smith, 2009; Shaffer et al., 2020).

## 5 Conclusion

The results of the present study provide further support for the findings that genetic neurodevelopmental syndromes exhibit asymmetrical linguistic profiles with specific strengths and weaknesses that can be identified in their narratives. In the microstructure, specific weaknesses stand out in the DS profile, except for productivity of utterances, while specific strengths emerge in the WS profile, except for grammatical complexity (MLU). In the macrostructure, no differences were observed between the syndromes, but a particular strength of adolescents with DS in the global level of the scenarios, and a relative weakness of adolescents with WS in the detailed level of the events should be highlighted. Thus, in the DS group, weaknesses in microstructure did not parallel the relative pragmatic strength in the overall retelling of the story, while in the WS group, strengths in productivity (utterances and tokens) and lexical complexity (types) did not translate into a more detailed retelling. Evidence from the present study supports the hypotheses of uneven asymmetrical narrative profiles in the adolescents with Down syndrome and Williams syndrome, and of dissociations between microstructure and macrostructure, suggesting that they could be the result of atypical trajectories of development which have been reported in the wider literature.

The present study also yielded some results concerning the typically developing groups. As expected, in the adolescents with typical language development, microstructure and macrostructure were closely correlated; but this was not the case in the group of typically developing 3-year-old children, which suggests that the association between microstructure and macrostructure is achieved across typical development.

Furthermore, given the wide use of MLU in language acquisition studies, it is worthwhile to underscore that we found no relation between MLU and narrative macrostructure variables in the typically developing or syndromic groups, i.e., the structure of a story may be told equally with short or long sentences. Conversely, lexical diversity was the best predictor of the structure of the stories even in the early stages. These findings may also have methodological implications in relation to the controversial issue of selecting control groups in studies of neurodevelopmental disorders.

## Data Availability

The raw data supporting the conclusions of this article will be made available by the authors, without undue reservation.
